# A practical approach to improve the statistical performance of surface water monitoring networks

**DOI:** 10.1007/s10661-019-7475-3

**Published:** 2019-05-01

**Authors:** Niina Kotamäki, Marko Järvinen, Pirkko Kauppila, Samuli Korpinen, Anssi Lensu, Olli Malve, Sari Mitikka, Jari Silander, Juhani Kettunen

**Affiliations:** 1 Finnish Environment Institute, P.O. Box 35, FI-40500 Jyväskylä, Finland; 2 Finnish Environment Institute, Latokartanonkaari 11, FI-00790 Helsinki, Finland; 3 Department of Biological and Environmental Science, University of Jyväskylä, P.O. Box 35, FI-40014 Jyväskylä, Finland

**Keywords:** Monitoring, Water Framework Directive, Classification, Confidence, Chlorophyll, Phosphorus

## Abstract

The representativeness of aquatic ecosystem monitoring and the precision of the assessment results are of high importance when implementing the EU’s Water Framework Directive that aims to secure a good status of waterbodies in Europe. However, adapting monitoring designs to answer the objectives and allocating the sampling resources effectively are seldom practiced. Here, we present a practical solution how the sampling effort could be re-allocated without decreasing the precision and confidence of status class assignment. For demonstrating this, we used a large data set of 272 intensively monitored Finnish lake, coastal, and river waterbodies utilizing an existing framework for quantifying the uncertainties in the status class estimation. We estimated the temporal and spatial variance components, as well as the effect of sampling allocation to the precision and confidence of chlorophyll-*a* and total phosphorus. Our results suggest that almost 70% of the lake and coastal waterbodies, and 27% of the river waterbodies, were classified without sufficient confidence in these variables. On the other hand, many of the waterbodies produced unnecessary precise metric means. Thus, reallocation of sampling effort is needed. Our results show that, even though the studied variables are among the most monitored status metrics, the unexplained variation is still high. Combining multiple data sets and using fixed covariates would improve the modeling performance. Our study highlights that ongoing monitoring programs should be evaluated more systematically, and the information from the statistical uncertainty analysis should be brought concretely to the decision-making process.

## Introduction

Environmental monitoring is the cornerstone of evidence-based environmental management. The monitoring is usually based on traditional methods and well-established standards. For many existing water quality monitoring programs, sampling takes place at fixed sampling locations and is carried out at regular intervals. This approach is generally justified by the need for standard time series, but it can also produce data that is either too excessive or insufficient in time or space in the light of the assessment and management objectives (Levine et al. 2014). This together with the continuous need to produce data more cost-efficiently (Nygård et al. 2016) has raised the dual need for, firstly, evaluating the efficiency and sufficiency of the ongoing monitoring schemes and, secondly, estimating the confidence of the assessment products. The need for representative environmental monitoring programs has been encountered in European member states in the implementation of the Water Framework Directive (WFD; EC 2000). The WFD has aimed to increase the monitoring efforts, improve assessment methodologies, and intensify the management of waterbodies in EU member states (e.g., Heiskanen et al. 2004; Borja et al. 2008; Hering et al. 2010; Birk et al. 2012). The ultimate aim of the WFD is that river, lake, coastal, and transitional waterbodies should achieve a good ecological and chemical status. For this, the waterbodies have been classified into “High,” “Good,” “Moderate,” “Poor,” or “Bad” ecological status classes (and “Good” or “Failing to achieve good” chemical status classes). The ecological status is based on several biological quality elements that are especially sensitive to key pressures, such as (human induced) eutrophication and changes in physical habitats (Anonymous 2003a). Whenever the desired status is not met, plans for management measures aiming to improve the status have to be made. The reliability of the status assessment is crucial for cost-effective river basin management, and the managers need to be confident when making decisions whether or not to invest money for often expensive management actions. A waterbody incorrectly assessed as having a “less than good status,” while the status is in reality good leads to an unnecessary waste of resources and money on wrongly targeted management actions. Vice versa, a falsely assessed good status may result in no allocation of water protection resources, which may have other consequences to society. To address this sort of misclassification, WFD requires the member states to determine the precision and the confidence of the classification (Anonymous 2003b, Annex I). Therefore, the most dominant errors, sources of variation, in the status class indicators have to be identified and quantified.

Quantification of different variance sources and addressing the uncertainty in assessing biological quality elements is not a novel approach, but its implementation often lags behind in practice in designing aquatic monitoring. Therefore, any practical applications of the method could speed up the positive development of monitoring programs. Carstensen and Lindegarth (2016) presented a coherent and well-established framework for quantifying uncertainties in status assessment. They listed 18 different sources of variation that a waterbody can be subjected to, the sources including spatial and temporal variation and methodological uncertainty (e.g., errors due to sampling methods, instruments, analysts, and replications) (see also Carvalho et al. 2013). For example, the year-to-year and within summer variation in lakes have a considerable influence on the classification results of phytoplankton (Thackeray et al. 2013; Søndergaard et al. 2016) and macrophytes (Dudley et al. 2013). In addition, spatial variation between sampling sites in a waterbody or sampling occasions within a sampling site affects the uncertainty along with temporal variation, as was shown for marine phytoplankton communities (Dromph et al. 2013). Additionally, laboratory analysts and the water depth may introduce a significant source of error, as demonstrated with eelgrass shoot density in coastal environments (Balsby et al. 2013, Bennet et al. 2011), and for lake phytoplankton (Carvalho et al. 2013). As for rivers, the classification of benthic diatoms is affected especially by temporal variability (Kelly et al. 2009) and river macroinvertebrates are affected by spatial, temporal, and replicate variation (Clarke 2013).

This study aims to demonstrate how to concretely bring the information from uncertainty analysis to the decision-making process when improving the monitoring design and evaluating assessment outcomes. For this, (i) the uncertainty in the status classification is evaluated by estimating a set of temporal and spatial variance components. The effect of the sampling allocation within and between years and between sampling sites on the precision and confidence of the class metric at a waterbody level is assessed. In order to support wider implementation of the approach, (ii) we present clear steps to carry out the analysis. Moreover, based on analysis, (iii) we come up to the decision rules that policy makers and water managers can utilize when adapting the monitoring programs to provide more precise status assessments and thus to determine suitable management actions. The approach is demonstrated for two widely used indicators for eutrophication, chlorophyll-*a* (chla) and total phosphorus (TP). The concentration data for these are available from 272 Finnish coastal, lake, and river waterbodies for the period 2006–2013.

## Materials and methods

### Study areas and data

The analysis in this study is based on chla and TP data from the most regularly monitored waterbodies in Finland. The minimum requirement for a lake and a coastal site to be selected for the dataset was that the samples of chla were taken at least 14 times during the period 2006–2012. A river waterbody was included in the analysis if there were a minimum of 60 TP sampling occasions in 2009–2012. The seasonal window follows the Finnish classification system (Aroviita et al. 2012; Andersen et al. 2016) applying the late summer periods for chla in lakes and coastal waters, and the whole year for TP concentrations in rivers (Table 1). In the dataset, 71% of lake waterbodies and 61% of coastal waterbodies were represented with one sampling site, the rest of the waterbodies at least two sites. In contrast, the river waterbodies were mainly (90%) represented by one sampling site.

**Table 1:** Overview of the data in different water categories and the number of waterbodies, sampling sites, and observations

	Lakes	Coastal waters	Rivers
Metric	chla (μg/l)	chla (μg/l)	TP (μg/l)
Sampling depth (m)	0–2	0–5	≤ 1
Period	2006–2012	2006–2012	2006–2012
Months	Jun–Sep	Jul–1st week of Sep	Jan–Dec
Waterbodies (no.)	161	38	73
Sampling sites (no.)	257	67	115
Samples (no.)	6707	1448	10,406

The analyzed waterbodies represent broadly different waterbody types in Finland (Table 2). The definition of the types follows the Common Implementation Strategies (CIS) of the Water Framework Directive (Anonymous 2003a and 2003c). The typology factors for rivers include altitude, catchment size, and its geology, and for lakes surface area, altitude, mean depth, humic substances (estimated by water color), and retention time (Pilke 2012). The Finnish classification system includes established reference conditions for each national lake and river type. The coastal types are based, among other aspects, on geographical location, salinity, mean depth, and the mixing conditions of the water (Kangas et al. 2003, Schenewski and Wilgat Schernewski and Wilgat 2004, Pilke et al. Pilke 2012). Basically, the coastal types are divided into inner, middle, and outer coastal waters/archipelagos, the inner types being generally shallower than the water in the outer types (Table 2). Summer time surface salinity ranges from below 3 practical salinity units (psu, ‰) in the Bothnian Bay to around 6‰ in the south-western archipelagos (incl. the Archipelago Sea) where it decreases towards the eastern Gulf of Finland. In general, the outer coastal waterbodies are larger in size than the innermost waterbodies, which are also usually more affected by river waters than the outer coastal types.

**Table 2:** Names, characteristics, and sample sizes of Finnish lake, river, and coastal waterbody types used in the analysis (*A* = area, *z* = mean depth, Sal = salinity, CA = catchment area)

Type and name	Characteristics	Waterbody (*n*)	Sample (*n*)
Vh; small and medium clear water lakes	*A* < 4000 ha; color < 30 mg Pt/l; *z* ≥ 3 m	10	408
Kh; medium-sized humic lakes	*A* 50–4000 ha; color 30–90 mg Pt/l; *z* ≥ 3 m	16	730
SVh; large clear water lakes	*A* > 4000 ha; color < 30 mg Pt/l	30	1912
Sh; large humic lakes	*A* > 4000 ha; color ≥ 30 mg Pt/l	16	867
Rh; very humic lakes	Color > 90 mg Pt/l; *z* ≥ 3 m	9	203
MVh; shallow clear water lakes	Color < 30 mg Pt/l; *z* < 3 m	4	168
Mh; shallow humic lakes	Color 30–90 mg Pt/l; *z* < 3 m	12	380
MRh; shallow very humic lakes	Color > 90 mg Pt/l; *z* < 3 m	15	363
Lv; lakes with a very short retention time	Retention time 10 days or less	5	209
Rr; nutrient-rich lakes	Naturally rich in nutrients	22	909
Rk; calcium-rich lakes	Naturally rich in calcium	2	91
Ss; Gulf of Finland inner archipelago	Sal 3.9–5.1 psu, *z* 9–11 m, *A* 85–117 km^2^	3	71
Su; Gulf of Finland outer archipelago	Sal 4.2–5.3 psu; *z* 22–26 m, *A* 423–579 km^2^	5	225
Ls; southwestern inner archipelago	Sal 5.0–5.9 psu; *z* 3–16 m; *A* 24–50 km^2^	9	205
Lv; southwestern middle archipelago	Sal 5.6–5.9 psu; *z* 6–33 m; *A* 29–424 km^2^	6	339
Lu; southwestern outer archipelago	Sal 5.9–6.2 psu; *z* 10–24 m; *A* 56–14,992 km^2^	7	287
Seu; Bothnian Sea outer coastal waters	Sal 5.5–5.6 psu; *z* 10–14 m; *A* 172–482 km^2^	1	14
Mu; Quark outer archipelago	Sal 3.3–5.1 psu; *z* 12–16 m; *A* 255–1076 km^2^	2	50
Pu; Bothnian Bay outer coastal waters	Sal 0.8–3.2 psu; *z* 8–15 m; *A* 69–1337 km^2^	5	257
Pt; small peatland rivers	CA < 100 km^2^; > 25% of CA peatland; col. > 90 mg Pt/l	1	119
Psa; small rivers in regions with clay soils	CA < 100 km^2^	2	270
Kt; medium-sized peatland rivers	CA 100–1000 km^2^; > 25% of CA peatland; col. > 90 mg Pt/l	8	628
Kk; medium-sized mineral soil rivers	CA 100–1000 km^2^; < 25% of CA peatland; col. < 90 mg Pt/l	4	510
Ksa; medium-sized clay soil rivers	CA 100–1000 km^2^	14	2451
St; large peatland rivers	CA 1000–10,000 km^2^; > 25% of CA peatland; col. > 90 mg Pt/l	15	1644
Sk; large mineral soil rivers	CA 1000–10,000 km^2^; < 25% of CA peatland; col. < 90 mg Pt/l	5	616
Ssa; large rivers in regions with clay soils	CA 1000–10,000 km^2^	7	1082
Est; very large peatland rivers	CA > 10,000 km^2^; > 25% of CA peatland; col. > 90 mg Pt/l	6	1089
ESk; very large mineral soil rivers	CA > 10,000 km^2^; < 25% of CA peatland; col. < 90 mg Pt/l	11	1997

### Indicator means and variance components

The chla and TP mean values and metric uncertainty are derived from a statistical, mixed effects model. In the linear mixed effects model, the indicator variable is expressed as a linear sum of fixed and random variables (Pinheiro and Bates 2000; Zuur et al. 2009). The fixed part of the model describes the mean value, and the random part includes the spatial and temporal variance components. For the status class modeling, it is assumed that the log-transformed waterbody indicator concentrations (*y*_*ijkl*_) are normally distributed with a mean *μ* and variance *σ*^*2*^ denoted as *log(y*_*ijkl*_*)~N(μ, σ*^*2*^*)*. The log-transformation is used to linearize the relationship and to normalize the right skewed response variables. This usually normalizes the residuals, which is the pre-assumption in the linear mixed modeling. A single measurement *l* from a year *i*, month *j*, and sampling site *k* can be expressed as a sum of the overall mean *μ* (expected value) and the components of random variation. For simplicity, it is assumed that all the variability is random:

1 $$ \log \left({y}_{ijkl}\right)=\mu +{\mathrm{year}}_i+{\mathrm{month}}_j+{\mathrm{site}}_k+{\varepsilon}_{ijkl} $$

The interannual variation (year_*i*_), monthly variation (month_*j*_), and the between sampling sites variation (site_*k*_) are assumed to be independent and normally distributed as year_*i*_~(0,${\sigma}_{\mathrm{year}}^2$), month_*j*_~풩(0,${\sigma}_{\mathrm{month}}^2$), and site_*k*_~풩(0,${\sigma}_{\mathrm{site}}^2$). Correspondingly, for the residual variation, *ε*_*ijkl*_~(0,${\sigma}_{\varepsilon}^2$). As the overall mean and the variance components are unknown, they are estimated from the data using a statistical mixed effects model. The analyses were conducted using the R statistical programming language (R Development Core Team, 2016) package lme4 (Bates et al. 2015). For comparing the precisions of the status class means in different waterbodies, the relative standard error (RSE%) was calculated. It is defined as the ratio of the estimated standard error to the estimated mean $\mathrm{RSE}\%=\Big({\widehat{\sigma}}^2/\widehat{\mu \Big)}100$. A small RSE% indicates precise metric mean classification and high error indicates more variation around the mean. In practice, the water manager makes the final decision about the accepted level of uncertainty.

### Confidence of a class

The estimated mean and uncertainty, thus the classification result, defines a normal probability distribution. The shape and the spread of the distribution show the range of indicator values that the waterbody data have taken. Following the standard notations and probability calculations described in other WFD contexts (e.g., Kelly et al. 2009, Lindegarth et al. 2013), the confidence of the status class can be calculated using the normal distribution. The probability (*p*_*i*_) of observing an indicator value *x* or better on the condition that the true mean quality (*μ*) is equal to the class boundary (퐿_i_) is expressed as 푝_i_ = *Pr*(푋 ≥ 푥 | 휇 = 퐿_i_) = 1 – Φ [(푥 −퐿_i_)/ *σ*] where Φ denotes the cumulative normal probability and the *σ* is the standard error of the mean calculated with the statistical model. This leads to the confidence of the class “High” being 100(1–*p*_High_), the confidence of the class “Good” being 100(*p*_Good_–*p*_Moderate_), the confidence of the class “Moderate” being 100(*p*_Moderate_–*p*_Poor_), the confidence of the class “Poor” being 100(*p*_Poor_–*p*_Bad_), and the confidence of class “Bad” being 100*p*_Bad_. These probabilities add up to 100%. The confidence of the metric class depends on the position of the metric status class boundaries (퐿_i_). The width and the position of the individual metric’s status class boundaries (as concentrations) vary between water categories and types.

### Implications for the monitoring design

The data that were analyzed with the linear mixed effects model (Eq. 1) correspond to a sampling design, where each sampling site is revisited repeatedly. Therefore, the overall variation in Eq. 2 (*σ*^2^) can be expressed as a sum of the random components of variation (Cochran 1977, Clarke 2013, Carvalho et al. 2013, Carstensen and Lindegarth 2016). The finite population correction factors, 1 − *n*_year_/*N*_year_ and 1 − *n*_month_/*N*_month_, are needed as there is a finite number of years in the assessment period and within years. Here, the maximum number of years within the assessment period 2006–2012 (*N*_year_) is 7, maximum number of months (*N*_month_) for lake chla is 4 (Jun–Sep), for coastal chla 3 (Jul–1st week of Sep), and for river TP 12 (Jan–Dec). For waterbodies with only one sampling site, the sampling site variation (${\sigma}_{\mathrm{site}}^2$) cannot be estimated and it is therefore zero.

2 $$ {\sigma}^2=\frac{\sigma_{\mathrm{year}}^2\left(1-\frac{n_{\mathrm{year}}}{M{\mathrm{n}}_{\mathrm{year}}}\right)}{n_{year}}+\frac{\sigma_{\mathrm{month}}^2\left(1-\frac{n_{\mathrm{month}}}{M{n}_{\mathrm{month}}}\right)}{n_{\mathrm{month}}}+\frac{\upsigma_{\mathrm{site}}^2}{n_{\mathrm{site}}}+\frac{\sigma_e^2}{n_{\mathrm{year}}{n}_{\mathrm{month}}{n}_{\mathrm{site}}n} $$

Depending on the relative size of the variance components and using this formula, it is possible to choose the number of sampled years (*n*_year_), months (*n*_month_), sites (*n*_site_), or replicate samples (*n*) the way that the overall variance is minimized. This information can be used for decision making when evaluating the ongoing monitoring programs and planning more targeted ones.

## Results

### The overall uncertainty

**Fig. 1:**
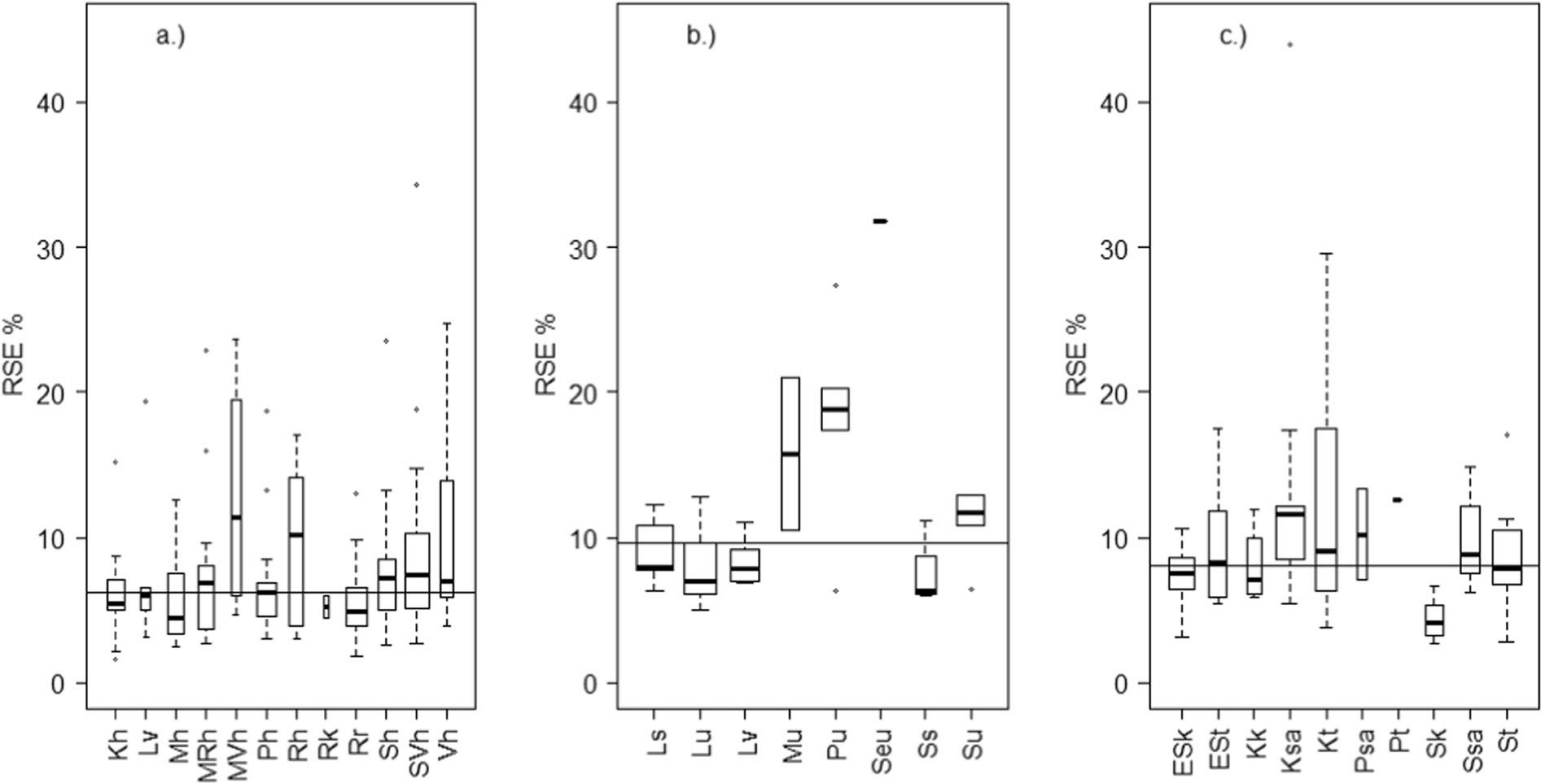
Total error (RSE%) of the mean metric for waterbody types (Table 2) in a.) lakes, b.) coastal areas, and c.) rivers. The box plots show the median, lower, and upper quartiles and outliers. The box widths are proportional to the number of observations in each waterbody type. For visualization, the widths denote the square roots of the number of observations. The median RSE% of each water category is denoted as a vertical line (6% for lakes and 10% for the coastal chla values, and 8% for the river TP)

The overall metric uncertainty was estimated for all the 272 waterbodies as the relative standard error of the mean (RSE%). The RSE% for an individual lake waterbody’s chla mean varied from a minimum of 2% to a maximum of 34%, and for coastal waterbodies from 5% to 32%. For rivers, the RSE% of TP means varied from 3 to 44%. The median RSE% for chla means in coastal waterbodies was 10% and in lakes 6%, and for TP means in rivers 8% (Fig. 1). For lakes, the smallest mean uncertainty (5%) was in waterbodies belonging to shallow and medium-size humic lakes (Mh, *n*_WB_ = 12; Kh, *n*_WB_ = 16), and nutrient-rich lakes (Rr, *n*_WB_ = 22). The median RSE% was low also for very calcareous lakes but only two waterbodies of this type were included in the analysis. The highest median error occurred in shallow, low-humic lakes (MVh, 11%, *n*_WB_ = 4), and very humic lakes (Rh, 10%, *n*_WB_ = 9). For coastal waterbody types, the RSE% varied from the median of 6% for waterbodies in the Gulf of Finland inner archipelago (Ss, *n*_WB_ = 3) to 19% for the Bothnian Bay outer coastal waters (Pu, *n*_WB_ = 5) and to 32% for a one waterbody in the Bothnian Sea outer coastal waters (Seu). The median TP uncertainty between river types varied from 4% of large rivers in regions with mineral soils (Sk, *n*_WB_ = 5) to 13% of a small peatland river (Pt) and to 12% of medium-sized rivers in regions with clay soils (Ksa, *n*_WB_ = 14). Rivers that are located in regions with mineral soils (types ESk, Sk, Kk) seemed to have smaller levels of uncertainty than rivers in peatland (types ESt, Kt, Pt, ST) or clay soils (Ksa, Psa, Ssa).

**Fig. 2:**
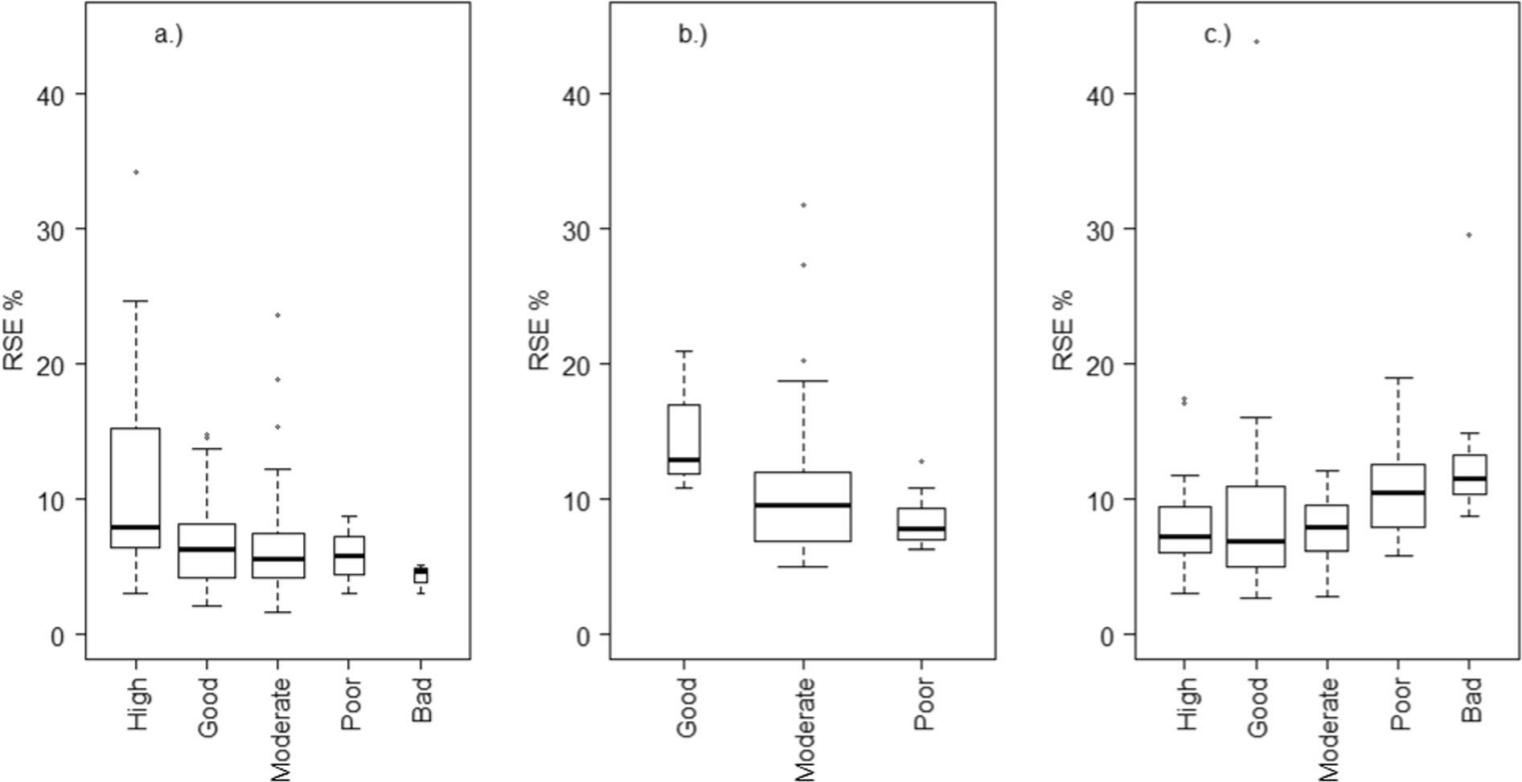
Total error (RSE%) of mean metric for estimated status classes within the waterbodies of a.) lakes (chla class), b.) coastal areas (chla class), and c.) rivers (TP class). The box plots show the median, the lower, and upper quartiles and outliers. The box widths are proportional to the number of observations in each status class. For visualization, the widths denote the square roots of the number of observations.

For chla in lakes*,* when the status class was estimated as high, the total error (RSE%) was also high and the variation between waterbodies was high (Fig. 2). When shifting to poor and bad classes, the RSE% and also the variation between waterbodies decreased. There are no coastal waterbodies with “High” or “Bad” chla class, but an increase in RSE% along the improvement of status class can be observed as well. However, for river TP, the variance seems to be generally higher in “Poor” and “Bad” classes than in the “High,” “Good,” or “Moderate.”

### Variance components

**Fig. 3:**
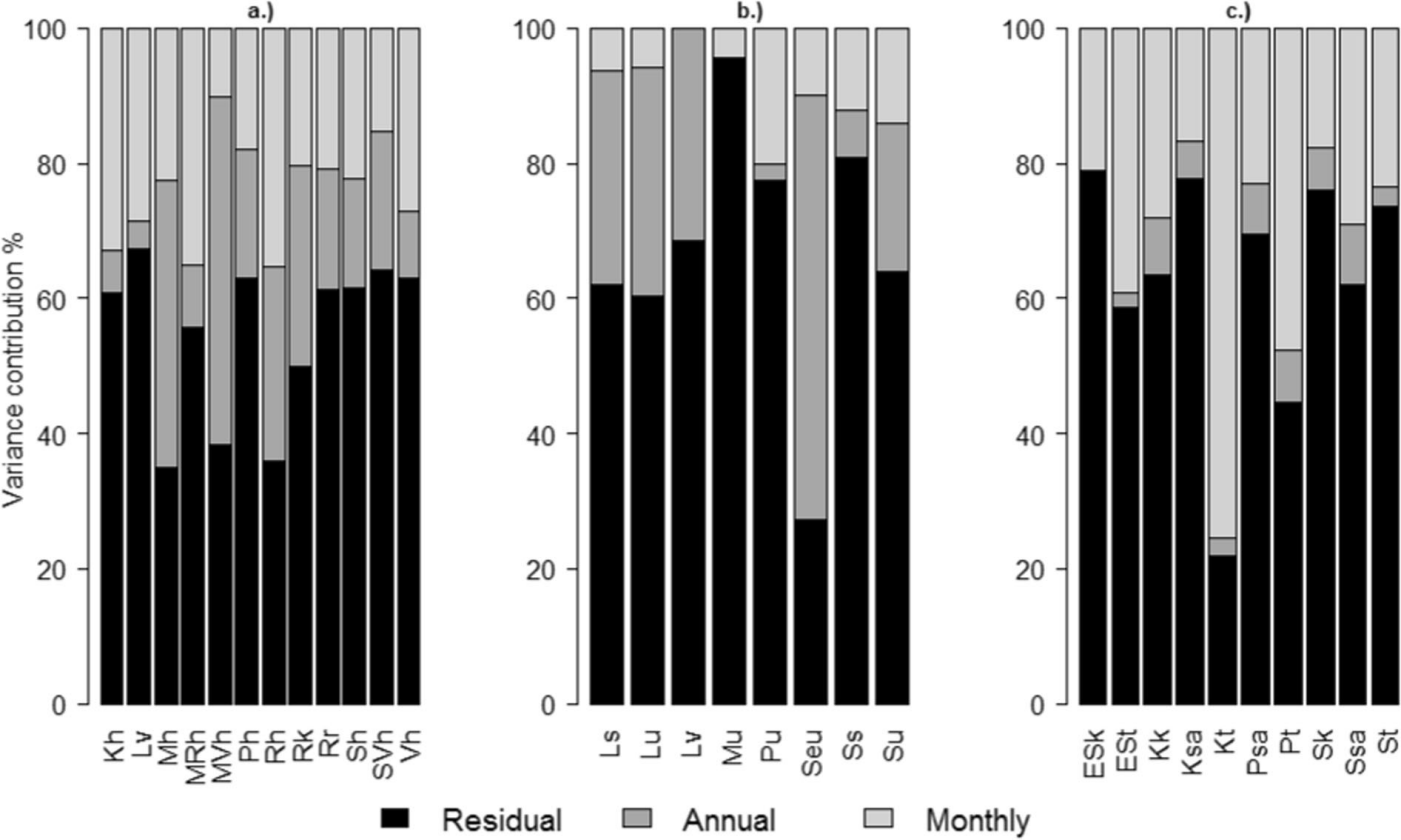
Relative sizes of residual and temporal (annual, monthly) variance estimates for a.) lake and b.) coastal chla and c.) river TP in different waterbody types

For waterbodies with one sampling site, the overall uncertainty consisted of random temporal variances between years and months and the unexplained residual variation. The overall variance contribution varied considerably between single waterbodies and between water category types. In general, the residual variation was the most dominant (Fig. 3). For chla in waterbodies with one sampling site, and only temporal variation estimated, the median residual variability was 61% for lakes and 66% for coastal waterbodies. For rivers, the residual variation was up to 67%. For lake types, and especially for coastal types, the interannual chla variation was usually higher than the between-month variation. For river TP, the monthly variation was a more dominant error source than the interannual variation.

**Fig. 4:**
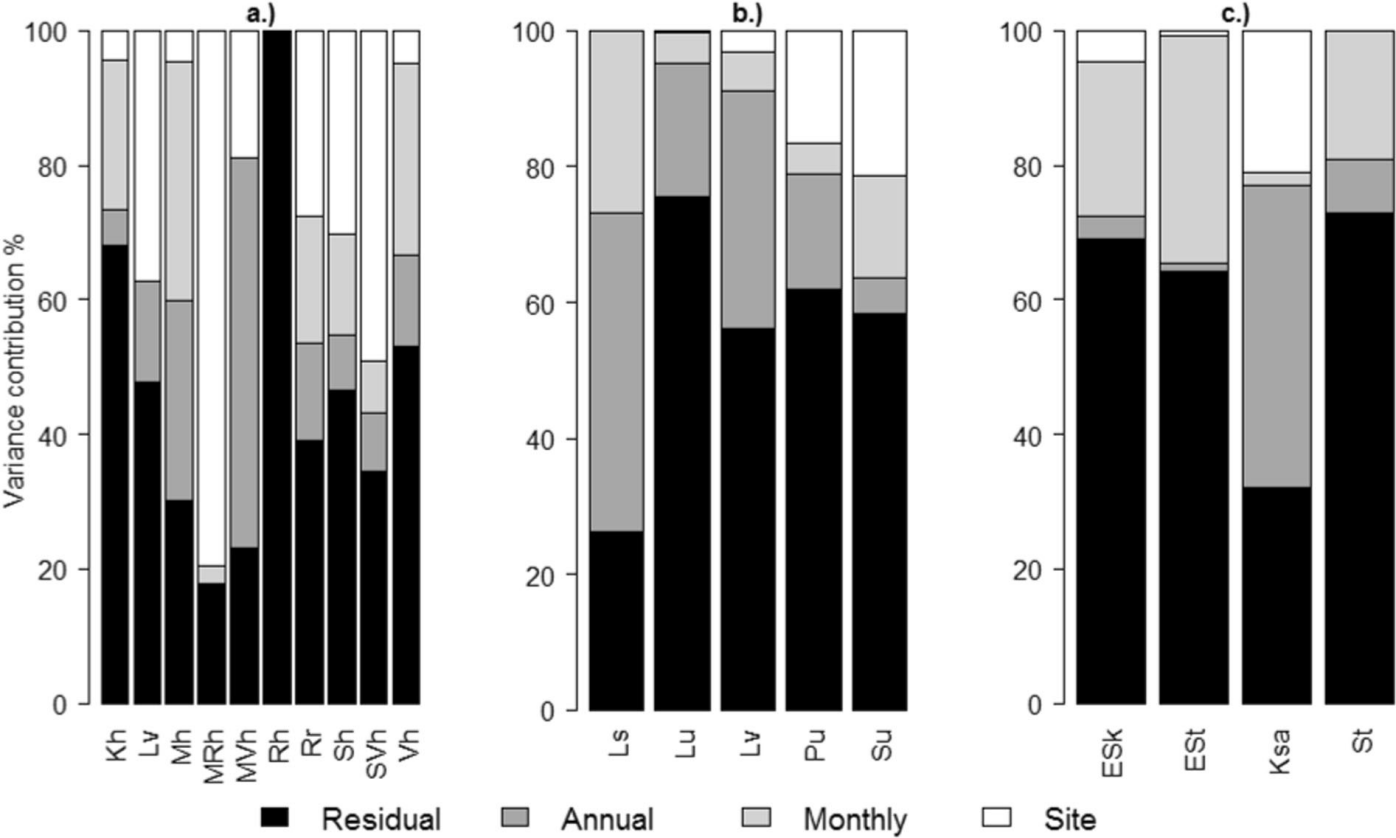
Relative sizes of residual, temporal (annual, monthly), and spatial (sampling site) variance estimates for a.) lake and b.) coastal chla and c.) river TP in different waterbody types

If there was more than one sampling site within a waterbody, in addition to monthly and annual variance components, between sampling sites variance was estimated. For lake waterbodies, this spatial variation was in many cases the most dominant source of variation (Fig. 4). For example, for large, low-humic lakes, the between-site variation covered almost half of the total variation in average. For coastal areas, the Bothnian Bay outer coastal waters (Pu) and the Gulf of Finland outer archipelago (Su), the site variation seemed to account a considerable part of the overall variation. The same hold for the medium-sized rivers in regions with clay soils (Ksa).

### Confidence of a class

**Fig. 5:**
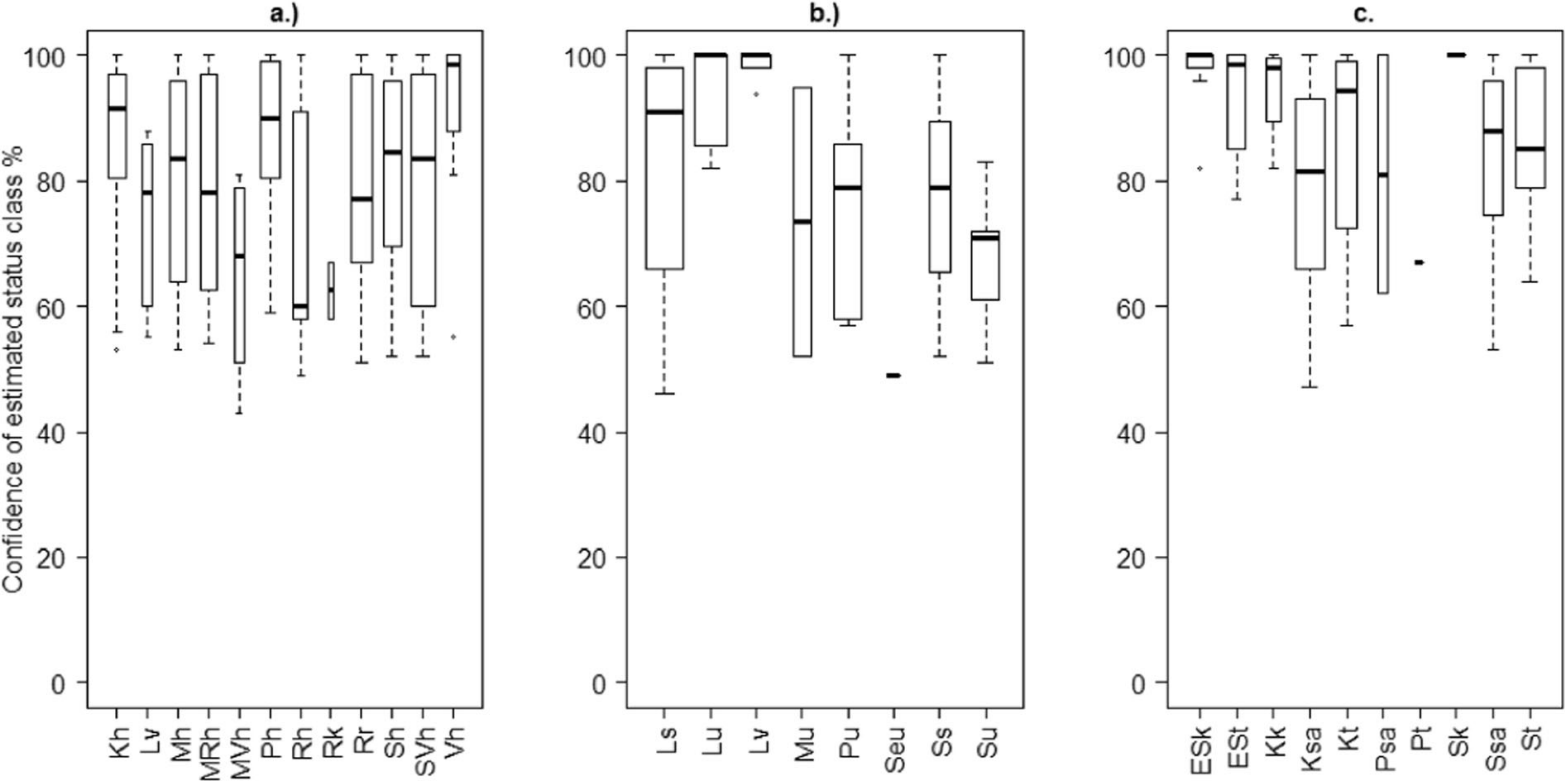
Distributions of the status class confidence (%) within the estimated status classes in a.) lakes (chla), b.) coastal areas (chla), and c.) rivers (TP). The box plots show the median, the lower, and upper quartiles and outliers. The box widths are proportional to the number of observations in each status class. For visualization, the widths denote the square roots of the number of observations.

The estimated status class confidence, denoted as the probability of the metric mean class, varied in lake waterbodies from 43 to 100%, in coastal waterbodies from 46 to 100%, and in river waterbodies from 47 to 100%. For over 63% of the waterbodies, the status class confidence was at least 80%. The status class confidence was generally high in all water categories: for river waterbodies, the median of the TP class confidence was as high as 96%. For the chla means for coastal waterbodies, it was 88% and for lakes, this stood at 83% (Fig. 5). However, the confidence of a class varied between waterbody types, ranging from ca. 50 to 100% in all water categories (Fig. 5). While the very large rivers (ESk and ESt) had a high confidence level, the rivers with clay/silt soil (Ksa, Ssa, Psa) had greater variation in the confidence of the status classifications. The confidence of the classification in the coastal waterbodies varied between and within the types but no clear pattern based on typology could be detected. Relatively, the status class confidence was greatest for the waterbodies in the middle Archipelago Sea (Lv), where 8 out of 13 waterbodies reached a confidence level exceeding 90%. Considering the south-western types altogether covering the Archipelago Sea and the western Gulf of Finland (Ls, Lv, Lu), around half of the studied waterbodies (12 out of 21 waterbodies) achieved a confidence level of more than 90%. However, the variation in the confidence was significantly greater near the coast (Ls), where the waterbodies represent smaller areas and are affected by river waters. The lake waterbodies showed similar variation in confidence between the lake types. The highest confidence level with the smallest variation was within the low-humic (Vh) lake type.

**Fig. 6:**
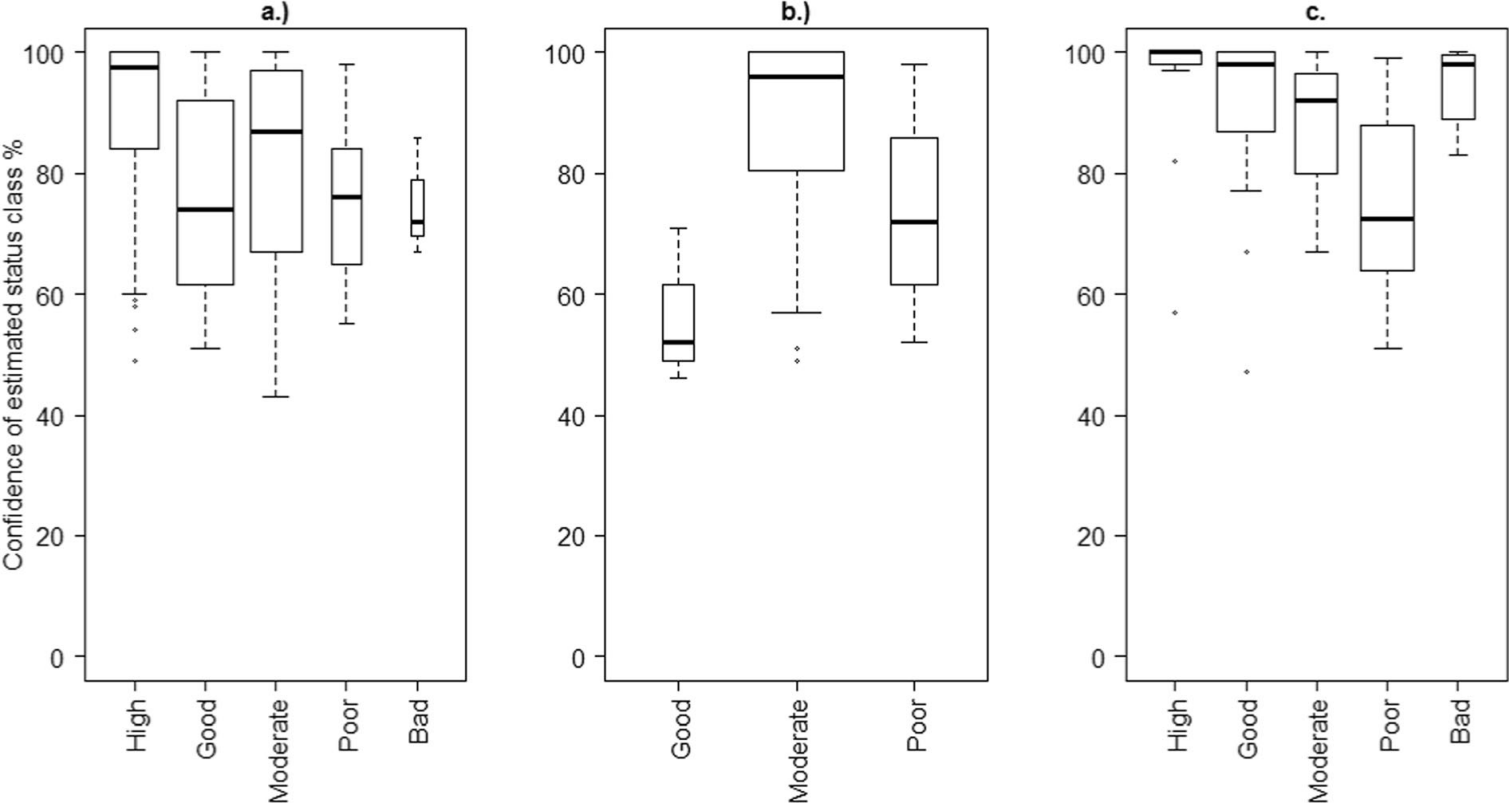
Distributions of the status class confidence (%) within different status classes for a.) lakes (chla class), b.) coastal areas (chla class) and c.) rivers (TP class). The box plots show the median, the lower and upper quartiles and outliers

The degree of confidence for the mean status class varied within and between the water categories (Fig. 6). For lakes and rivers, the class “High” showed the highest confidence. In lakes, the greatest variation in confidence occurred for the classes “Moderate” and “Good,” whereas, in rivers, this was for the class “Poor.” “Bad” and “High” status classes did not exist in coastal waterbodies, and the confidence level for the “Good” class was the lowest.

### Implications for the monitoring design

**Fig. 7:**
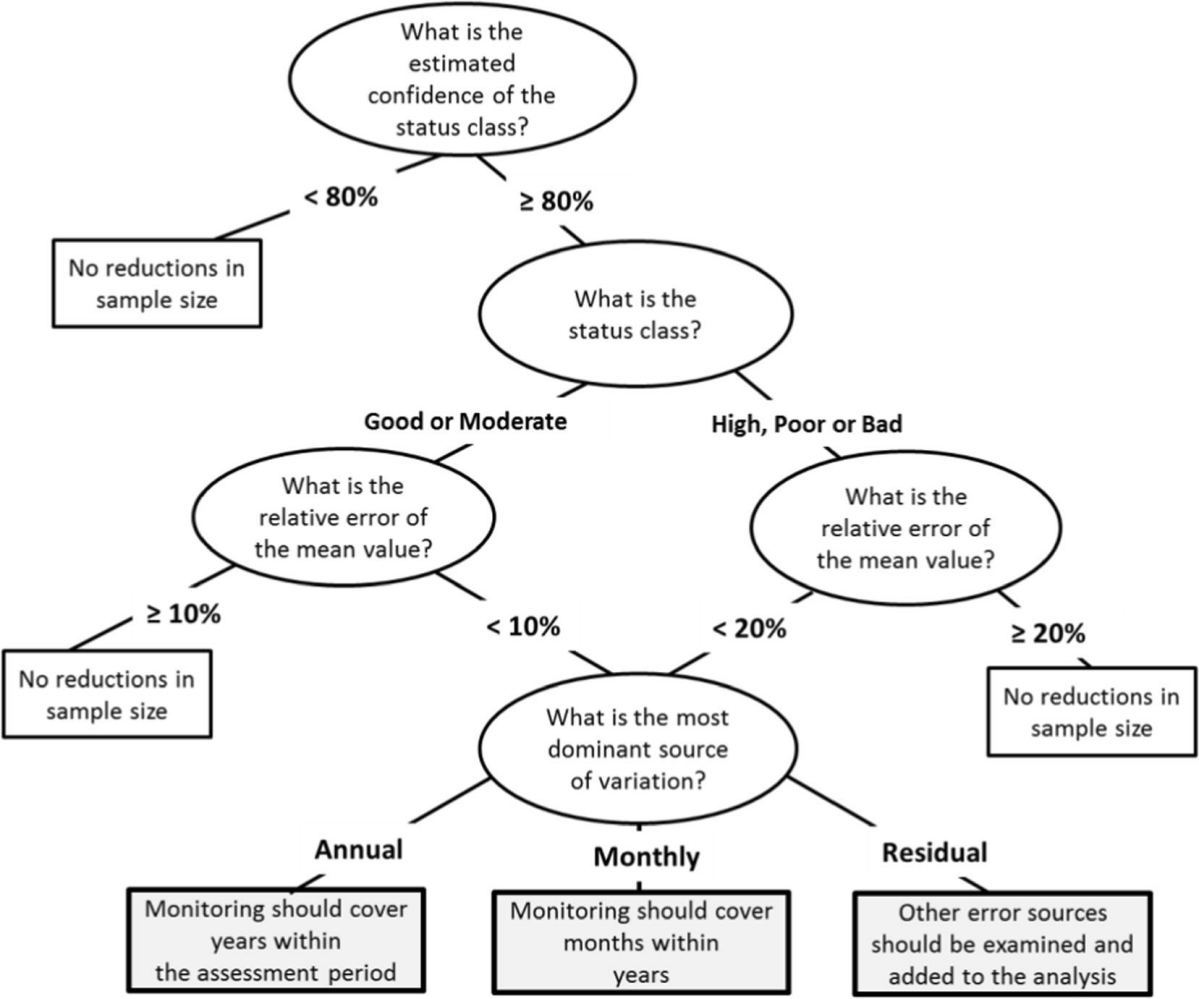
An example of a decision chain for aiding how to allocate the waterbody level monitoring effort optimally in temporal scale

In close collaboration with policy makers (regional environmental authorities from the Centres of Economic Development, Transport and the Environment), we created simple decision rules that help in the decision-making process in monitoring design for classification purposes. Here, the decision rules are illustrated in the case where the sampling in time could be reallocated (Fig. 7). First, the probability of the status class with the highest probability is expressed as the confidence of a class. A sufficient confidence was set to 80% which was seen as a reasonable target of the confidence level for the most intensively monitored waterbodies. If the status class confidence was lower than 80%, more monitoring effort would be needed in order to improve the status class confidence. However, if the status classification falls near the class boundary, increasing the sampling frequency would not help (Clarke and Hering 2006). For such waterbodies, the resources should be guided towards management methods. When the confidence was estimated to be higher than 80%, the status class determines the next step. If the status class was “Good” or “Moderate” and the RSE% higher than 10%, a more precise status classification is needed. This is because the “Good” and “Moderate” class limit has the greatest implication for the decision whether or not to start management measures. For the extreme classes (“High,” “Bad,” “Poor”), the RSE% higher than 20% leads to a need for more sampling effort. However, for waterbodies classified as “Good” and “Moderate” and the RSE% estimated less than 10%, or “High,” “Bad,” or “Poor” classes with RSE% less than 20%, the sampling design might produce even unnecessarily precise status class. Based on the most variance components, the sampling could be targeted more optimally in time. If the most dominant source of variation was the between-years variability, then all years of the assessment period should be covered. On the other hand, if the monthly variation was the largest source, the most important months within a year should be covered, but the monitoring could be performed, e.g., every third year (rotating panel design). Finally, if the unknown residual variation was the highest source of uncertainty, the other unknown error sources should be further examined or a fixed covariate should be added to the model.

Based on the simple decision rules, it was possible to identify those Finnish waterbodies where the sampling effort could be reduced or reallocated without losing the precision and thus the confidence in the status classification. For 40% (108/272) of the studied Finnish waterbodies, the status class confidence was over 80% and the precision of the mean metric was high (the RSE% was under 20% or 10% depending on the status class (Table 3). Therefore, the data from these waterbodies were identified as producing sufficiently or even unnecessarily precise status class metric mean. On the other hand, for almost 60% of the waterbodies, the confidence of the status class estimates was low and therefore, reductions in sample size were not recommended. From the intensively monitored lake waterbodies 69% (111/161) and from the coastal waterbodies 68% (26/38) were lacking sufficient sampling effort for reliable status class mean assessment using chla. For rivers, the TP class metric was usually more precise, which is seen in the amount of waterbodies with sufficient sampling (63%).

**Table 3:** Result of the statistical decision chain analysis (Fig. 7) showing the number of Finnish lakes, coastal, and river waterbodies for which the sampling effort is sufficient or should be increased in the light of precise metric mean. Expressed in lakes, coastal, and river waterbodies and in chla or TP status classes

	More sampling needed	Sampling sufficient	Total
Lakes	111	50	161
High	12	27	39
Good	34	12	46
Moderate	48	6	54
Poor	10	3	13
Bad	7	2	9
Coastal	26	12	38
Good	3		3
Moderate	17	9	26
Poor	6	3	9
Rivers	27	46	73
High	1	16	17
Good	8	12	20
Moderate	5	6	11
Poor	12	6	18
Bad	1	6	7
Total	165	108	272

## Discussion

Quantifying and ultimately reducing the indicator uncertainty and its components have been viewed as a way towards achieving more reliable and transparent status assessments (Birk et al. 2012). Further, the decision makers would benefit from knowing not just the average status but also the probabilities of each status class and they could therefore more intuitively select the appropriate management measures (Reyjol et al. 2014; Hering et al. 2010). The standard scientific approach for expressing uncertainty is done through probabilities (Sigel et al. 2010). However, quantification of uncertainty in terms of probabilities has not been very broadly assessed in the context of WFD classification even though there is a need to support methods taking uncertainty into account. Clarke (2013) introduced a framework and decision-making tool to calculate the probability of a waterbody belonging to each status class according to the WFD. Examples of probabilistic tools to quantify status class confidence using a Bayesian modeling framework include single metrics such as a fish index in rivers (Marzin et al. 2014) and phytoplankton in lakes (Kotamäki et al. 2015) and coastal waters (Fernandes et al. 2012). We have used a probabilistic distribution to account for the naturally high variation in two status class metrics, river TP and chla in lakes and coastal waters. This reduces the risks of misclassification and helps water managers to make decisions more confidently. Although the importance of quantifying the ecological indicator’s uncertainty in assessing the status is understood, the issue is still rarely addressed and even less implemented in practice (Carstensen and Lindegarth 2016).

In this study, the classification uncertainty was estimated for 272 Finnish waterbodies for two status class metrics. For lake and coastal waterbodies, we used chla, which is a cost-effective and robust metric reacting rapidly to eutrophication pressure (Phillips et al. 2008). However, this sensitivity also makes the chla metric highly variable (Lyche-Solheim et al. 2013, Carvalho et al. 2013). The result of this study suggested that the uncertainty, expressed as a relative standard error of the chla mean, was higher in coastal waterbodies than in lakes. As for Nordic rivers, chla is an unsuitable metric (Annex X in Mischke 2016); TP was used instead. It should however be noted that TP is a supporting quality element in the classification of rivers and does not alone fulfill the requirement of the WFD ecological status assessment. The use of TP instead of yet relatively scarcely available ecological metrics in most Finnish rivers can be justified by statistical connection proved between the biological indicators and phosphorus concentrations (e.g., Paisley et al. 2011). Additionally, TP in rivers is intensively and regularly sampled, which allows for feasible estimation of its variance components. The TP class uncertainty in Finnish river waterbodies varied in our study from 2 to 44%. The highest uncertainties were observed in rivers with clay-dominated catchment soils.

On average, the coastal chla assessment showed slightly larger errors (10%) than the lakes (6%). This is in line with the fact that coastal ecosystems, especially in the northern Baltic Sea, are morphometrically and hydrodynamically complex, and hence, the spatiotemporal variations are expected to be high (Kauppila 2007; Borja et al. 2013). For lakes, it was difficult to draw any clear conclusions about the variation between different national lake types. On average, the lowest uncertainties were within the shallow and medium-sized humic lakes and the highest uncertainties on the other hand within the shallow, low-humic and very humic lakes. The river TP means were classified with a high degree of confidence and a low error. However, the overall uncertainty varied substantially between the river types. In general, the size and the soil of the catchment area have been shown to be significant factors producing differences in TP variation (Vuorenmaa et al. 2002).

Comparing the different uncertainty components, the unexplained random variation (residual error) was often the most dominant source of variability. For rivers, the residual variation was high (up to 67%), which is in accordance with the general understanding that the riverine nutrient concentrations are highly correlated with other sources than just temporal variability (such as the weather and water flows). The results of this study suggest that, in coastal waterbodies with a single sampling site, the annual variation was a larger source of uncertainty than the between-month variation. The intra-annual variation already reduces as the classification scheme accounts only for a short period of time (between July and early September). For river TP, the largest source of uncertainty was the between-month variation and this occurred in all river types which is in line that riverine nutrients are highly variable (Edwards and Withers 2008; Tattari et al. 2017). In coastal waterbodies, which in general are very dynamic systems, the site variation did not occur as dominant as would have been expected. Especially concerning the outer waterbodies, this is, firstly, because of the relatively sparse monitoring network, and, secondly, because of the patchiness of phytoplankton biomasses in open and coastal marine waters (see Reinart and Kutser 2006; Harvey et al. 2015). In contrast, the between-site variation was a large source of uncertainty for the lakes with more than a one sampling site. Our results contradict the results of the sampling experiment done for selected European lakes (Thackeray et al. 2013) where increasing the number of open water sampling stations visited, or the number of samples collected at each station, did little to improve the precision of ecological assessments based upon the phytoplankton metrics.

If the variance components had been disregarded and the error of the mean had been calculated from the data using the sample standard deviation, the uncertainty would have been grossly underestimated. When testing this for a single lake, Lake Lentua, the summertime monthly variation was the most prominent (59%) and the estimated standard error was 24%. However, the error was only 10% when calculated from the sample data and ignoring the temporal variance. This highlights the fact that, to gain realistic uncertainty estimates for status classifications, the different sources of variation have to be accounted for. If the indicator variance is wrongly determined (usually underestimated, as discussed), or not determined at all, it gives a false impression of the confidence and precision of the indicator. This can lead to insufficient judgements when making decisions about the management actions.

The surface water monitoring programs in Finland and in other EU member states have been evolving with the requirements of the WFD. However, the long traditions and the large number of waterbodies have led to challenges for planning and optimizing the monitoring schemes. Additionally, the ongoing pressure to reduce or optimize monitoring resources calls for systematic examination for better monitoring allocation. However, it is impossible to change the monitoring scheme to become more adaptive unless there is a sound scientific foundation to rely on. Besides expressing the status class uncertainty as probabilities, the information of the different sources of uncertainty can be utilized also in planning and optimizing monitoring programs (Gitzen et al. 2012). Although a lot of research has been conducted on the quantification of the different variance components, less research has been carried out to apply this knowledge to improve the monitoring designs. The need for improving the monitoring programs was acknowledged in the context of evaluating the success and challenges obtained from the implementation process of the WFD (Birk et al. 2012; Hering et al. 2010). In assessing coastal status based on macrophyte index, Cavallo et al. 2016 concluded that there are alternative ways to perform the monitoring in respect to its spatial and temporal coverage without losing the confidence of the classification. Similar approaches have been conducted by Thackeray et al. (2013) and Carvalho et al. (2013) for lake phytoplankton and Clarke (2013) for river macroinvertebrates.

When the most dominant sources of uncertainty and total error have been identified and quantified with variance components, this information can be used for allocating the sampling effort so that the overall uncertainty is reduced. The practical guidance demonstrated in this study and in some earlier studies (e.g., Clarke and Hering 2006; Clarke 2013; Carstensen and Lindegarth 2016) helps the decision maker to enhance the monitoring resources. The sample size directly affects the standard error of the mean and the probability distributions, thus the confidence of the status class. In practice, the results suggest that, for coastal waterbodies, the confidence levels can be improved by ensuring annual sampling, whereas, for lakes, higher confidence would require more sampling sites within a lake waterbody. Following the suggested practical steps towards improving the monitoring design, one should analyze the variance components on the level of waterbody types or waterbodies and not only rely on generalizations. The confidence of classification depends also on which status class the metric mean assigns the waterbody to. Our results show that, for many waterbodies, the confidence of estimating good and moderate status was low. Previous studies have shown that, near the class level boundaries, the confidence is low (Kelly et al. 2009). Also, the width of status class has an effect on the confidence of a class. For narrower classes, the uncertainties and the probability of misclassification are higher (Clarke 2009, Kelly et al. 2009 and Mascaró et al. 2013). The higher variation in the middle classes (moderate and good) stems also from the wide range of natural conditions within these classes that can either favor or hamper chla and TP levels. More emphasis should be placed when operating especially within the critical good and moderate status classes. Equally important would be to start identifying the unknown sources of uncertainty in status assessments to further improve the reliability of the classification results.

The WFD-related phytoplankton sampling frequency is typically 1–6 times per year in the Nordic lakes (Poikane 2009, Carvalho et al. 2013) and 2–18 times per year in Finnish coastal waters (Korpinen 2014). For river TP at least fortnightly-monthly sampling is recommended in the WFD guidance (Anonymous 2003b). However, the minimum monitoring frequencies quoted in the Directive may not be adequate or realistic, especially for transitional and coastal waters due to higher variability and heterogeneity of most marine systems (Anonymous 2003b). Our analysis was conducted using data from the most frequently monitored Finnish waterbodies and according to WFD guidance. Even though part of the spatiotemporal variation could be covered, it was still impossible to estimate many of the possible uncertainty components from these data. For example, the spatial within waterbody variance, which can be high especially for chla, was impossible to derive from data with only one sampling site. In addition, longer datasets should be analyzed for filtering the possible trends from the time series. On the other hand, the studied metrics, chla and TP, do not represent the overall ecological status that is derived from several quality elements composed of multiple metrics. In the WFD classification, the individual metric values are scaled to Ecological Quality Ratios (EQR) to allow comparability between different assessment methods. EQR implicitly includes information about the reference conditions; therefore, it might lead to added (and unknown) uncertainty. In addition, aggregating the data to EQR level might add bias as has been discussed, e.g., in Carstensen and Lindegarth 2016. Hence, the status assessment in this study refers only to a computational, sample-based class of chla or TP, which are single metrics of phytoplankton quality element or supporting element of ecological classification. The statistical methods described here are applicable to other biological variables, such as macrophytes and phytobenthos, as well, but the monitoring of these quality elements is even sparser than for phytoplankton. The reason for this is partly because of the WFD monitoring is established for multiple purposes and objectives requiring numerous variables to be measured in different spatial and temporal scales.

## Conclusions

This study is among the few making an effort to estimate systematically the precision and confidence of status class metrics and using this information for reallocating the sampling effort of an ongoing monitoring program. Here, we presented a practical method to analyze the variance components that build up the uncertainty of status assessment and the probability of reporting the correct status class. Our results showed that, for many waterbodies, the overall uncertainty was not well captured by the year-to-year, monthly or sampling location variations, but the largest variance component was often the residual variation. This indicates that some important sources of uncertainty were left ignored. In order to identify these, one should include more explanatory variables in the model as presented earlier, e.g., by Carstensen and Lindegarth (2016) and Malve et al. (Malve 2007). Frequency and coverage of monitoring designs should be systematically and iteratively evaluated with objectives that serve the river basin management planning. Moreover, in the future, the monitoring programs should combine different data sources, including not only the traditional water sampling but also the satellite data and automatic sensors. Combining such data can be implemented using spatiotemporal interpolation and Kalman filtering techniques (Cressie and Wikle 2011). This would provide more information for the assessments of the different sources of uncertainty. Especially, the spatial coverage and variation, which turned out to be significant source of classification uncertainty, would be better accounted for.
